# Diastolic Function Evaluations by Tissue Doppler Imaging in Patients With Mitral Valve Prolapse

**DOI:** 10.4021/cr256w

**Published:** 2013-03-08

**Authors:** Rezvaneh Salehi, Elnaz Javanshir, Elgar Enamzadeh

**Affiliations:** a Dept. of Cardiology, Cardiovascular Research Center, Tabriz University of Medical Sciences, Tabriz, Iran

**Keywords:** Mitral valve prolapse, Tissue Doppler imaging, Echocardiography, Diastolic dysfunction

## Abstract

**Background:**

Tissue Doppler imaging (TDI) is a new echocardiographic imaging technique that is believed to be superior to older conventional techniques in assessing abnormalities of cardiac function in many conditions affecting the heart. There are very limited data regarding the role of TDI in evaluating patients with mitral valve prolapse (MVP). Current study evaluates diastolic function in patients with MVP by this method.

**Methods:**

From November 2009 to April 2011, one hundred and ten adults matched for age and sex and without structural heart disease were studied in two groups, with and without MVP, at Madani Heart Center, Tabriz, Iran. TDI was performed at the basal-lateral wall and S, E, E’, A, and A’ velocities, as well as the E/A and E’/A’ ratios were measured. Mitral annular systolic velocity and early diastolic (E’) velocities were measured lateral corner of mitral valve in apical 4-chamber view.

**Results:**

Patients with MVP had higher left atrium volume (42.31 ± 10.47 vs. 35.19 ± 9.15 cm3; P < 0.001) and deceleration time (186.70 ± 26.00 vs. 176.89 ± 20.36 ms; P = 0.03). Diastolic dysfunction, although seemed more prevalent in MVP group (14.54%) than normal subjects (5.45%), the difference was not statistically significant between groups (P = 0.11).

**Conclusion:**

Left atrial volume and deceleration time of mitral valve inflow was significantly higher in MVP which could be indicative of early stages of diastolic dysfunction in patients with MVP. However, larger follow-up studies are required to evaluate clinical significance of our findings in these patients.

## Introduction

Mitral valve prolapse (MVP) is defined echocardiographically as single or bileaflet prolapse of 2 mm or more, beyond the long-axis annular plane, with or without leaflet thickening [1, 2]. The prevalence is estimated at 2-3%, and it is equally distributed between men and women [3]. MVP is associated with a genetic component [4] which may cause histological abnormalities of valvular tissue or geometric disparities between the left ventricle and mitral valve.

Mitral valve prolapse is a fairly heterogeneous disease according to its natural course. Populational studies show that more than half of patients with MPV are asymptomatic and usually show a benign course of the disease. Their overall morbidity and mortality is similar to the general population. However, in the remaining portion of cases, MVP may be associated with severe cardiovascular complications such as progressive mitral regurgitation, arrhythmias, heart failure, and increased risk of infective endocarditis. Finally, 7.8% of the patients with MVP require mitral valve surgery [5].

Historically, physical examination and transthoracic echocardiography (TTE) have been the diagnostic reference standards for MVP. However, physical findings such as the classic mid-to-late systolic click with a high-pitched late systolic murmur have low specificity, and TTE occasionally does not yield sufficient information for a diagnosis [1, 6].

Tissue Doppler imaging (TDI) is a newer echocardiographic imaging technique that is believed to be superior to older conventional techniques in assessing abnormalities of cardiac function in many conditions affecting the heart [7]. Today by the use of TDI subtle derangements in systolic and diastolic function can be diagnosed. Myocardial velocity measurements with TDI in systolic and diastolic phases are indicative of ventricular function in systolic and diastolic cycle. These TDI parameters are minimally affected by heart flow, so it is possible to evaluate systolic and diastolic function at the same time [8].

There are very limited data regarding the role of TDI in evaluating patients with MVP [9]. The aim of current study is to evaluate diastolic function in patients with mitral valve prolapse by TDI, as a new technique in cardiac imaging.

## Methods

The study was carried out at Madani Heart Center, Tabriz/Iran, from November 2009 to April 2011. A total of 110 adult subjects, aged 18 years and older, were studied in two groups. The first group consisted of 55 consecutive patients with newly diagnosed MVP, who met the inclusion and exclusion criteria. The second group consisted of 55 apparently normal controls with no known valvular disease, which were referred because of palpitation. Both groups were matched for age (27.65 ± 8.37 and 28.02 ± 7.31 years; P = 0.81) and sex (male; 38.18% vs. 34.54%; P = 0.69).

Patients with associated cardiac disorders (for example, other valvular lesions, coronary artery disease, cardiomyopathies, and left ventricular ejection fraction < 40%) and patients with systemic hypertension, significant mitral regurgitation, and atrial fibrillation were excluded from the study. Those receiving beta blockers and other drugs with influence on left ventricle diastolic function were also excluded.

All patients gave written informed consents and the study was approved by our local Ethics Committee.

### Mitral valve prolapse diagnosis

Mitral valve prolapse (MVP) is an abnormal movement of one or both mitral valve leaflets (morphologically normal-appearing, or redundant and thickened) into the left atrium during systole. The diagnosis of MVP was made by physical examination (mid-to-late systolic click and a systolic murmur at auscultation) and echocardiography. The echocardiographic diagnosis of mitral valve prolapse was based on the parasternal long-axis view. Mitral valve prolapse was defined as systolic displacement (> 2 mm) of one or both mitral leaflets into the LA, below the plane of the mitral annulus [10].

All echocardiographic studies were performed by one experiences echocardiographist. To define inter observer variability, echocardiograms of 15 patients with MVP and 15 control normal subjects were reviewed by another expert that reported the same findings.

Echocardiographic studies were performed Vivid 7 Dimension (General Electric Healthcare Company, Milwaukee, WI, USA).

### Tissue doppler measurements

Myocardial velocities were measured on-line using a standard pulse wave Doppler technique. Color-coded images were acquired during a breath hold over two consecutive cardiac cycles using low velocity, high-intensity myocardial signals at high frame rate (0.150 MHz). TDI of the mitral annulus was obtained from the apical 4-chamber view. A 1.5 mm sample volume was placed sequentially at the lateral and medial mitral annulus. All Doppler signals were recorded with a chart recorder set at 100 mm/s. The average of 3 end-expiratory cycles was used. Pulmonary venous flow was obtained using the right upper pulmonary vein in the apical four-chamber view. The measurements obtained were S-wave (systolic forward flow), D-wave (diastolic forward flow) and A-wave (atrial reversal). Analysis was performed for Mitral inflow measurements included peak early (E) and peak late (A) flow velocities, the E/A ratio, the systolic (S’) and the early (E’) and late diastolic velocity (A’), the systolic filling fraction (S/D) and the deceleration time of early mitral flow velocity (DT) (time interval of peak E-wave velocity to its extrapolation to the baseline). These variables were analyzed individually, as the average of the medial and lateral annulus, and as the maximum of the medial and lateral annulus.

LA volume was measured from standard apical 2- and 4-chamber views at end-systole. The left atrial volume was calculated using the formulae [11]: LV volume = 0.85 × (A1*A2)/L

Where A1 is left atrial area in the apical four-chamber view, A2 is left atrial area in the apical two-chamber view, and L is orthogonal (vertical) left atrial dimension in the apical four chamber view.

Comprehensive Doppler and two-dimensional features including E/A ratio, LA volume, DT, E/E’ ratio and A were used to assess LV diastolic function. Three grades of diastolic dysfunction have been defined. Grade I (E/A < 0.8, DT > 200 ms) is referred to as relaxation abnormality. Grade II (E/A = 0.8-1.5 > 1, DT = 160 - 200 ms) is called pseudo-normalization. Grade III is referred to decrease in relaxation time decreases and restrictive filling (E/A ≥ 2 and DT < 160 ms) [12].

### Statistical analysis

Continuous data with normal distribution are given as mean ± standard deviation, otherwise as median. Mean differences for continuous variables between groups were examined by the independent Student t-test. Chi-square test or Fisher exact test if applicable) was used for categorical variables. A P*-*value of 0.05 or less was considered significant. All calculations were done with SPSS for Windows (SPSS Version 16, SPSS Inc., Chicago, Illinois).

## Results

Complaints of patients in MVP group were palpitation (36.4%), chest pain (25.5%), and anxiety (3.6%). The rest were referred with the combination of above mentioned complaints. Auscultatory findings in these patients were increased S1 (16.4%), systolic click (14.5%) and systolic murmur (3.6%). Most patients (45.5%) had increased S1 sound and systolic clicks together, and 7.3% had a combination of these findings. However, 12.7% had normal cardiac auscultation.

Comparison of TDI measurements between the two groups (Table 1) revealed that patients with MVP had higher left atrium volume (42.31 ± 10.47 vs. 35.19 ± 9.15 cm^2^; P < 0.001) and deceleration time (186.70 ± 26.00vs.176.89 ± 20.36 cm/s; P = 0.03).

**Table 1:** Tissue Doppler Echocardiographic Parameters

Variables	MVP group	Normal group	P value
Left ventricle diameter (cm)	4.50 ± 0.43	4.35 ± 0.39	0.053
Left atrium diameter (cm)	2.73 ± 0.32	2.66 ± 0.32	0.3
Left atrium volume (cm^2^)	42.31 ± 10.47	35.19 ± 9.15	< 0.001
Mitral annulus diameter (mm) (4 chamber view)	27.84 ± 3.30	27.79 ± 2.75	0.55
Mitral annulus diameter (mm) (2 chamber view)	27.56 ± 3.22	27.55 ± 2.60	0.97
E (cm/s)	81.02 ± 18.93	81.20 ± 14.14	0.95
A (cm/s)	58.76 ± 14.63	59.51 ± 12.62	0.78
E’ (cm/s)	15.36 ± 4.14	16.45 ± 3.97	0.37
A’ (cm/s)	8.89 ± 3.62	8.69 ± 2.21	0.73
E/A	1.45 ± 0.47	1.41 ± 0.34	0.59
E’/A’	2.08 ± 1.09	2.03 ± 0.96	0.79
E/E’	5.58 ± 2.67	5.33 ± 1.33	0.39
S (cm/s)	22.38 ± 2.72	10.78 ± 3.24	0.29
Pulmonary vein systolic flow velocity (cm/s)	51.85 ± 9.59	55.09 ± 9.26	0.07
Pulmonary vein diastolic flow velocity (cm/s)	51.56 ± 13.24	49.33 ± 12.77	0.37
S/D	1.06 ± 0.30	1.18 ± 0.36	0.07
Left ventricle ejection fraction (%)	62.82 ± 5.10	63.05 ± 5.21	0.44
Deceleration time (ms)	186.70 ± 26.00	176.89 ± 20.36	0.03

E: peak early mitral inflow velocity; A: peak atrial mitral inflow velocity; S: systolic forward flow; D: diastolic forward flow; E’: early diastolic myocardial velocity; A’: late diastolic myocardial velocities.

Diastolic dysfunction, although seemed more prevalent in MVP group (14.54%) than normal subjects (5.45%), the difference was not statistically significant between groups (P = 0.11). Diastolic dysfunction was mild (grade 1) in 5 cases and moderate (grade 2) in 3 cases of MVP group. All three cases in normal group had mild diastolic dysfunction.

## Discussion

TDI findings in our study showed that left atrial volume and deceleration time were significantly higher in patients with MVP. There was also a nonsignificant trend towards larger left ventricle diameter in these patients.

Scampordanis et al [13] for the first time showed some grades of systolic contractile dysfunction in MVP patients. Other studies with 2D and M-mode echocardiography have also shown abnormal systolic function in these patients [14, 15]. However, in our study we observed no systolic dysfunction in patients with MVP. This could be due to different patient populations with respect to age and severity of prolapse. The study findings by Dagdeviren et al [16] are in accordance with this presumption. They found a distinct spectral Doppler pattern of mid- and basal posterior and lateral walls consistent with spikes on systolic velocities. These spikes were noticed in 65% of patients with MVP. The MVP patients with spikes had larger mitral annulus diameters and higher amounts of maximal leaflet displacement when compared with those without spikes.

In our study, mean deceleration time was significantly higher in the MVP group which is indicative of impaired diastolic relaxation in these patients. On the other hand, left atrial volume was also higher in this group, which once more is in favor of the probability of diastolic dysfunction. Nishimura et al [17] have also reported the presence of diastolic dysfunction in MVP patients. In another study, Zampoulakis et al [18] reported some degree of diastolic dysfunction, particularly after exercise, in the individuals with MVP. They observed that the mean S-wave and Am wave at rest was higher in subjects with MVP compared to that of the control groups, whereas after exercise these were higher in the control group. The mean Em wave and Em/Am ratio at rest was higher in the control individuals both at rest, and after exercise. These findings were indicative of less effective contractile response to exercise compared to healthy individuals [18].

We did not find these differences between our study groups. In Zampoulakis’s study, patients with MVP were older than the control group [18]. Our study groups were matched according to age and sex, which could be the reason for these differences. Also we studied subjects only at rest.

In conclusion, among parameters studied, left atrial volume and deceleration time were significantly higher in patients with MVP, which could be indicative of early stages of diastolic dysfunction in these patients. However, larger follow-up studies are required to evaluate clinical significance of our findings in these patients.
